# Crosstalk between the endocannabinoid and mid-brain dopaminergic systems: Implication in dopamine dysregulation

**DOI:** 10.3389/fnbeh.2023.1137957

**Published:** 2023-03-16

**Authors:** Berhanu Geresu Kibret, Ana Canseco-Alba, Emmanuel S. Onaivi, Ephrem Engidawork

**Affiliations:** ^1^Department of Biology, College of Science and Health, William Paterson University, Wayne, NJ, United States; ^2^Direction de Investigacion, Instituto Nacional de Neurologia y Neurocircirugia “Manuel Velasco Suarez”, Mexico City, Mexico; ^3^Department of Pharmacology and Clinical Pharmacy, School of Pharmacy, College of Health Sciences, Addis Ababa University, Addis Ababa, Ethiopia

**Keywords:** endocannabinoid system, dopamine, schizophrenia, drug addiction, sexual motivation

## Abstract

Endocannabinoids (eCBs) and the expanded endocannabinoid system (ECS)-“endocannabinoidome”, consists of the endogenous ligands, eCBs, their canonical and non-canonical receptor subtypes, and their synthesizing and metabolizing enzymes. This system modulates a wide range of body functions and acts as a retrograde signaling system within the central nervous system (CNS) by inhibition of classical transmitters, and plays a vital modulatory function on dopamine, a major neurotransmitter in the CNS. Dopamine is involved in different behavioral processes and contributes to different brain disorders—including Parkinson’s disease, schizophrenia, and drug addiction. After synthesis in the neuronal cytosol, dopamine is packaged into synaptic vesicles until released by extracellular signals. Calcium dependent neuronal activation results in the vesicular release of dopamine and interacts with different neurotransmitter systems. The ECS, among others, is involved in the regulation of dopamine release and the interaction occurs either through direct or indirect mechanisms. The cross-talk between the ECS and the dopaminergic system has important influence in various dopamine-related neurobiological and pathologic conditions and investigating this interaction might help identify therapeutic targets and options in disorders of the CNS associated with dopamine dysregulation.

## Introduction

The discovery of the endocannabinoids (eCBs) as a family of lipid molecules, and the advances in the identification of other putative receptors, congeners of eCB mediators, and their enzymes formed a complex and expanded endocannabinoid system (ECS) known as “endocannabinoidome” (eCBome; Cristino et al., 2020). The ECS is widely distributed in almost all human cells and tissues and exerts a multitude of cellular signaling mechanisms involved in the regulation of several functions including retrograde signaling in the central nervous system (CNS) by modulating classical neurotransmitters and many types of synaptic plasticity (Jonsson et al., 2006). The isolation of Δ^9^-tetrahydocannabinol (Δ^9^-THC), the psychoactive component of cannabis, in 1964 (Gaoni and Mechoulam, 1964) laid a cornerstone of advances in cannabinoid research. However, the isolation of endogenous cannabinoid compounds, and the characterization of cannabinoid receptors (CBRs) and their chromosomal localization (Onaivi et al., 2002; Joshi and Onaivi, 2019) heralded the explosion in cannabinoid research.

Dopamine (DA) is a major CNS neuro-messenger involved in different behavioral processes and contributes to several neuropsychiatric disorders (Iversen et al., 2010). The dopaminergic pathway is composed of a group of cells called dopaminergic neurons, whose cell bodies are found in the three distinct midbrain nuclei: retrorubral field (A8), substantia nigra pars compacta (SNc, A9), and ventral tegmental area (VTA, A10; Hillarp et al., 1966). Tyrosine hydroxylase (TH) is the enzyme responsible for the synthesis of DA, which is released from both somatodendritic and axonal compartments following neuronal depolarization (Carlsson et al., 1958; Andén, 1967; Besson et al., 1969).

Previous studies provided data and neuroanatomical evidence for the functional interactions between the ECS and dopaminergic systems in the activity of the rat basal ganglia motor circuit (Julian et al., 2003). ECS modulation of DA function in other brain circuits including the mesocorticolimbic have been demonstrated (Covey and Yocky, 2021; Oleson et al., 2021), but the mechanism of DA release by eCBs requires further investigation, as eCB signaling in the nucleus accumbens (NAcc) facilitates goal seeking behavior. Interestingly, in a pre-clinical model, eCBs increase DA signaling in the NAc to facilitate goal-seeking behavior, thus eCB-based therapies may be investigated for motivational disturbance in addiction.

However, the multiple direct and indirect mechanisms of ECS modulation of dopaminergic and other neurotransmitter signaling is an important focus in understanding habit formation and reward pathways (Peters et al., 2021), not only in neuropsychiatric disturbances but in the emerging link between the eCBome and the microbiota-gut-brain axis (Di Marzo, 2020). In an analysis of rats perinatally exposed to THC and patients with schizophrenia spectrum disorders, Di Bartolomeo et al. (2021) provided evidence of crosstalk between transcriptional regulations of DA-CB1R interaction. Their preclinical data suggested that cannabidiol (CBD) treatment might normalize the perinatal THC-induced psychopathology by modulating the altered dopaminergic activity (Di Bartolomeo et al., 2021). Other studies provided evidence for a balance between direct and indirect modulatory mechanisms of the interaction between eCBs and DA. On the other hand, pre-clinical models of natural rewarding behavior, such as sexual behavior, have provided evidence for the role of the ECS in modulating its motivational component (Canseco-Alba and Rodríguez-Manzo, 2014; Rodríguez-Manzo and González-Morales, 2020), a psychobiological function in which DA is highly implicated (Pfaus et al., 1990).

Here, we review and discuss new knowledge highlighting the crosstalk between the expanding endocannabinoid and mid-brain dopaminergic systems. We reviewed recent advances in the era of global medical and recreational cannabis use and the impact and implication of the interaction between the ECS and dopaminergic system in CNS disorders associated with dopamine dysregulation. We highlight mitochondrial activity involving CB1Rs implicated in parvalbumin interneurons that may serve as biomarkers for diseases associated with dopaminergic dysregulation.

## The endocannabinoid system

The ECS is an intricate and complex ubiquitous signaling system than was previously thought. It is composed of cannabinoid receptors, an increasing family of diverse eCB lipid molecules, and their synthesizing and metabolizing enzymes (Scotter et al., 2010). More recently, the identification of other eCB long-chain fatty acid amides and esters with their metabolic enzymes, and putative CBRs constitute an expanded ECS that is called eCBome (Di Marzo, 2020). An emerging prominent role of the ECS is the regulation of cytokines and neurotransmitters, mainly DA, release from immune cells and neurons, respectively, which is crucial in the maintenance of homeostasis as an autoprotective response.

### Endocannabinoids

eCBs are lipid mediators that exert most of their numerous biological functions by binding and activating CBRs and non-CBRs. The two most studied eCBs are anandamide and 2-arachidonoylglycerol (Alhouayek and Muccioli, 2012). The synthesis of anandamide follows two steps. The first step is the conversion of arachidonic acid from the *sn*-1 position of phosphatidyl choline to form *N*-arachidonoyl phosphatidylethanolamine (NAPE) by transacylase. The second step involves the hydrolysis of NAPE to produce anandamide by phospholipase (Liu et al., 2008). An increase in intracellular calcium results in the activation of *sn*-1-specific diacylglycerol lipase-α and -β and subsequent conversion of arachidonoyl-containing diacylglycerol species to 2-arachidonyl glycerol (Hashimotodani et al., 2005).

Fatty acid amide hydrolase and monoacylglycerol lipase, respectively, are responsible for the metabolism of anandamide and 2-arachidonyl glycerol following cellular uptake. It has been also demonstrated that cyclooxygenase-2, 12- and 15-lipoxygenases oxidize anandamide and 2-arachidonyl glycerol. Metabolism is responsible for intracellular and extracellular signaling mechanisms of anandamide and 2-arachidonyl glycerol (Di Marzo, 2006).

### Cannabinoid receptors

CB1Rs and CB2Rs are the most commonly studied CBRs that are coupled to G-proteins. CB1Rs activation results in the inhibition of adenylyl cyclase (AC) *via* Gi and regulation of the inwardly rectifying potassium currents (Kir) in AtT-20 pituitary tumor cells in a pertussis toxin-sensitive manner through the βγ-subunits (Howlett, 2005). Activation of CB2Rs also inhibits the activity of AC enzymes. Both CB1Rs and CB2Rs receptors stimulate p42/p44 mitogen-activated protein kinase (MAPK) activity (Pertwee and Ross, 2002; Howlett, 2005). Activation of CB1Rs but not CB2Rs results in the release of inositol triphosphate and intracellular Ca^2+^ *via* Gi/o. Studies also showed stimulation of AC following low efficacy coupling of CB1Rs to the Gs G-protein (Glass and Felder, 1997; Turu and Hunyady, 2010).

CB1Rs, which are expressed in the basal ganglia nuclei, hippocampus, cerebellum, and neocortex, are the most prevalent GPCRs in the brain. They are found on GABAergic and glutamatergic neurons pre-synaptically (Scotter et al., 2010). CB2Rs are called peripheral receptors and they are highly found in immune cells (Munro et al., 1993; Galiègue et al., 1995; Brown et al., 2002) and were believed to be absent in the brain (Griffin et al., 1999; Brown et al., 2002; Poso and Huffman, 2008). Contrary to these earlier beliefs, a substantial body of research shows that CB2Rs are expressed in microglia and neurons in the brain stem, striatum, and hippocampus (Van Sickle et al., 2005; Onaivi et al., 2006; Brusco et al., 2008). Additionally, CB2Rs are expressed in the dopamine neurons in the CNS and are connected to drug addiction, synaptic plasticity, eating disorders, psychosis, depression, and autism spectrum disorders (Liu et al., 2017).

There are also non-CB1/CB2 receptors that are activated by eCBs. Due to the chemical similarity between anandamide and capsaicin, anandamide is also an agonist at the TRPV-1 receptor and this ability to stimulate vanilloid receptors appears to be governed by the state of activation of protein kinase A (PKA) and protein kinase C (PKC; Pertwee and Ross, 2002). Other orphan receptors, including GPR55 and GPR119 have been identified as putative CBRs. GPR55 is activated by several cannabinoids, and oleoylethanolamide mainly activates GPR119 (Overton et al., 2006). GPR55 is also a lysophosphatidylinositol receptor (Oka et al., 2007). The human caudate and putamen express the GPR55 gene, while the hippocampus, thalamus, pons, cerebellum, and frontal cortex do not. Specifically, the pancreas and gastrointestinal tract express GPR119 (Fredriksson et al., 2003). It serves as a glucose-dependent insulinotropic receptor and is coupled to Gαs in the pancreatic islets, which are cells that produce insulin (Chu et al., 2008). Cannabinoids have also been demonstrated to exert their effect through the activation of nuclear receptors, particularly peroxisome proliferator activated receptors (PPARs). Cannabinoids can directly activate PPARs and the latter are also indirectly modulated by receptors and enzymes that regulate the activity and metabolism of endocannabinoids. Conversely, the PPAR pathway is also modulating the EC system (Iannotti and Vitale, 2021; Lago-Fernandez et al., 2021). This regulation and modulation by a common ligand are important for the development of multitarget therapeutic strategies.

### Cannabinoid receptor signaling

CB1Rs and CB2Rs are coupled primarily to the Gi/o subtypes of G protein and receptor activation usually results in the inhibition of AC activity through the release of Giα isoforms (Rhee et al., 1998). Receptor heterodimerization might be responsible for the activation of both CB1Rs and dopamine DA2 receptors that resulted in subsequent activation of AC. The non-selective CBR agonist WIN-55212-2, but not other cannabinoids, has recently been reported to increase intracellular calcium in cultured hippocampal neurons and in human embryonic kidney 293 cells *via* coupling to Gq/11 proteins, despite the fact that direct evidence for the coupling of CB1Rs to Gq/11 was lacking for quite some time (Lauckner et al., 2005).

Different signaling cascades can be activated by CBR agonists in a distinctive manner. Gi and Go are found to be effectively coupled and activated by CB1Rs, but Go is the only G-protein that CB2Rs activate. Furthermore, agonist-selective G protein signaling is demonstrated by the fact that the effectiveness of a given agonist appears to rely on whether CB1Rs is coupled to Gi or Go (Glass and Northup, 1999; Prather et al., 2000). eCBs regulate synaptic transmission throughout the nervous system. The mechanism of eCB signaling in the nervous system is distinct from classical neurotransmitters. It occurs through a retrograde signaling mechanism, in which depolarization of the postsynaptic neuron induces the synthesis of eCBs, which are released and transported backwards to CB1Rs expressed primarily on the presynaptic terminal (Brady et al., 2012; Winters and Vaughan, 2021). This retrograde regulatory control of synaptic transmission by eCBs at CB1Rs can induce shor-term changes in synaptic strength and more complex long-term plasticity that are still poorly understood as reviewed by Winters and Vaughan (2021). The coupling of CB1R to the Gi/o G-protein produces a signaling cascade that controls calcium and potassium channels and ultimately inhibits further neurotransmitter release. Accordingly, eCB signaling modifies the effectiveness of transmission by promoting communication between postsynaptic and presynaptic neurons. The final result of eCB signaling depends on the characteristics of the involved cells because CB1R activation inhibits neurotransmission (Howlett, 2005).

Depolarization of postsynaptic hippocampal pyramidal cells or cerebellar Purkinje cells by increased intracellular calcium rapidly triggers the biosynthesis and release of eCBs. These are then believed to work through presynaptic CB1Rs to prevent the release of GABA or glutamate at the presynaptic level. It is interesting that depolarization-induced suppression of inhibition is anticipated to increase intense synaptic activity, but depolarization-induced suppression of excitation should provide a negative feedback mechanism for reducing high synaptic activity (Pertwee and Ross, 2002; Sidhpura and Parsons, 2011). Further research is warranted in understanding eCB-mediated plasticity in its multi-faceted complexity, regional and synapse specific differences of eCB retrograde signaling, and as bidirectional regulators.

### The endocannabinoidome

eCBs have molecular targets other than CBRs and TRPs, and these proteins are also targeted by other endogenous and exogenous substances. Moreover, eCBs have more than one set of metabolic enzymes and pathways, which they share with other mediators that may or may not be included in the definition of eCBs. As a result, the eCBome is emerging as an expanded definition, representing the ensemble of endocannabinoids, endocannabinoid-like mediators, and their several receptors and metabolic enzymes (Veilleux et al., 2019). The eCBome participates in multiple metabolic functions in health and disease, and aims to illustrate the interconnectedness of all systems in the body and how the ECS acts to maintain homeostasis in the body. There is also increasing knowledge of the functional roles of the gut-microbiome immune-brain axis with a link with the eCBome (Di Marzo and Silvestri, 2019). Insights from understanding the eCBome *per se* and its interaction with the gut-immune-brain axis could be used to unlock many medical mysteries that have eluded us for quite some time, including the neuropathogenesis of metabolic and neuropsychiatric disorders associated with dopaminergic dysfunction.

## The dopaminergic system

Dopamine is a monoamine catecholamine neurotransmitter and hormone involved in motor control, motivation, learning, and memory, and plays a major role in different neurodegenerative and neuropsychiatric disorders like Parkinson’s disease (PD), schizophrenia, and drug addiction (Iversen et al., 2010).

### Dopamine biosynthesis

DA is synthesized from tyrosine by the rate limiting enzyme TH, to produce L-DOPA, which is quickly decarboxylated by L-aromatic acid decarboxylase to DA (Andén, 1967; Scherman et al., 1988). Release of DA from vesicles happens from both somatodendritic and axonal terminal locations in a calcium-dependent manner (Besson et al., 1969). After release, DA acts through five subtypes of GPCRs (Lachowicz and Sibley, 1997). Finally, the Na^+^/Cl^−^ dependent plasma membrane dopamine transporter (DAT) transports DA in the extracellular space back into dopaminergic terminals, which can be stored in the vesicles or metabolized, or diffuses away from the synapse (Sotnikova et al., 2005).

### Dopamine receptors

DA subserves its function by activating five subtypes of receptors (D1-D5), which are abundant in the CNS. DA receptors are classified into two general classes: those that predominantly couple to Gαs/olf (“D1-like”, DA1 and DA5 receptors), and to Gαi/o (“D2-like”, DA2-DA4 receptors; Lachowicz and Sibley, 1997). Ligand binding studies in recombinant systems indicated affinity differences between the two classes of receptors for DA. Accordingly, the affinity of D2-like is higher by 10–100-fold than that of D1-like, suggesting that extracellular DA concentrations determine whether D1- or D2-mediated signaling predominates (Martel and Gatti McArthur, 2020). Looking at the abundance of receptors in the CNS, whilst D1 is the most abundant, D4 is the least abundant subtype of the receptor. D2 comes next to D1 and this is followed by D3 and D4. D1 receptors help regulate the development of neurons when bound by DA, explaining, at least in part, their abundance in the CNS. D1 and D5 receptors appear to have high density in brain structures involved in regulating reward, motor activity, learning, and memory. Apart from stimulating the cAMP pathway, D1 and D5 also stimulate the phosphoinositide pathway culminating in induction of intracellular calcium release and activation of PKC. Calcium modulates neurotransmitter release and PKC/PKA negatively modulates the renal Na+/K+ ATPase, thereby producing increased electrolyte excretion and renal vasodilation. Other dopamine signaling pathways, including modulation of the Akt-GSK3 and activation of the PAR4 pathways have also been reported (Hasbi et al., 2011). Moreover, heteromers of D1/D2 have been identified and are known to be coupled to the phosphoinositide pathway. D2, D3, and D4 receptors are expressed mainly in the striatum as well as the external globus pallidus, core of the nucleus accumbens, hippocampus, amygdala, and cerebral cortex. D2 is mainly involved in locomotion, attention, sleep, and learning and memory. D3 and D4 by and large, subserve similar functions, including cognition, impulse control and attention, and sleep. These receptors also affect the postsynaptic receptor-medicated extrapyramidal activity. D2-D4 receptors not only inhibit AC but also activate K+ channels *via* the βγ-subunits.

### Dopaminergic neurons

The A8–A17 neurons represent the main subgroups of dopaminergic neurons. These major groups are further divided into four main groups having a distinct set of physiological and psychological effects (Andrade, 2010). Groups A8–A10 make up the mesencephalic or midbrain dopaminergic neurons, groups A11–A15 make up the diencephalic dopaminergic neurons, and groups A16 and A17 make up the olfactory bulb and retinal dopaminergic neurons, respectively (Melis and Argiolas, 1995). The nigrostriatal, mesolimbic, and mesocortical pathways are three more sub-divisions of the mesencephalic or midbrain dopaminergic system, all of which originate from the A8–A10 cell groups. These dopaminergic neurons originate in a number of nearby mid-brain nuclei, such as -ld (nucleus A8 cells), the substantia nigra (nucleus A9 cells), and the ventral tegmental area (nucleus A10 cells).

**Figure 1:**
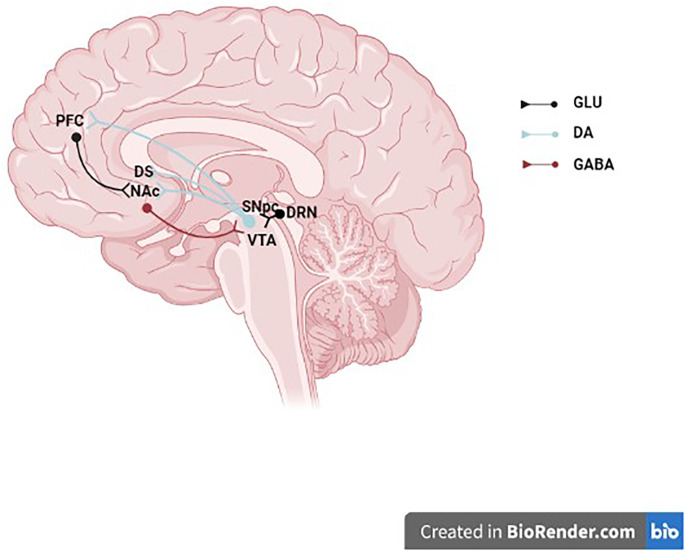
Midbrain dopaminergic neurons. The schematic illustrates a midbrain dopaminergic neurons projecting to the NAc, DS, and PFC and receiving synaptic inputs from a GLU DRN neuron and a GABAergic NAc neuron. GLU, glutamate; DA, dopamine; GABA, γ-aminobutyric acid; PFC, prefrontal cortex; DS, dorsal striatum; NAc, nucleus accumbens; SNpc, substantia nigra pars compacta; VTA, ventral tegmental area; DRN, dorsal raphe nucleus.

The dorsal striatum’s nigrostriatal dopaminergic pathway, which includes the caudate, putamen, and globus pallidus as well as neurons whose cell bodies primarily come from the SNc’s A9 group and, to a lesser extent, the VTA’s A10 neurons, are crucial for controlling and coordinating locomotor activity (Ungerstedt, 1976). With fewer neurons coming from the A8 and A9 groups than the nigrostriatal pathway, the majority of the neurons that make up the mesolimbic dopaminergic pathway project to the NAcc, amygdala, and olfactory tubercle (Figure 1). The mesolimbic dopaminergic pathway, commonly known as the “reward pathway” in the brain, has been linked to reward and pleasure in addition to its function in the control of affect, mood, and locomotor activity. The orbitofrontal, medial, dorsolateral, and cingulate regions of the prefrontal cortex (PFC) are among the regions that receive projections from the mesocortical dopaminergic system (Abi-dargham and Moore, 2003). According to several studies, social behavior, working memory, attention, and executive function are all influenced by the mesocortical dopaminergic neurons (Bubser and Schmidt, 1990; Sawaguchi and Goldman-Rakic, 1994; Floresco and Magyar, 2006). Overall, these DA neurons also spread within GABA neurons, thus establishing local connections with adjacent neurons, including the glutamatergic neurons (Zhang et al., 2014). The dual transmission is associated with the regulation of both GABA and glutamate neurotransmitters induced by eCB retrograde signaling (Laksmidewi and Soejitno, 2021).

**Figure 2:**
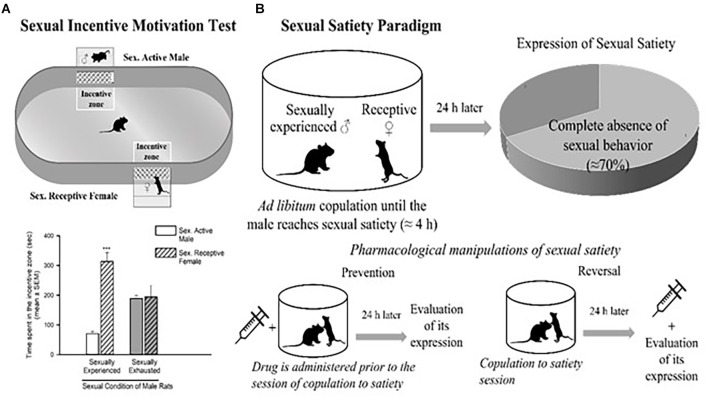
Sexual incentive motivation test **(A)** and sexual satiety paradigm **(B)**. Sexual incentive motivation is measured as the time spent by the male rat in the incentive zones (receptive female or male) of the test field. Sexually experienced males will spend more time in the incentive zone of the sexually receptive female (dashed bars) in comparison with the male (empty bars). Sexually satiated males will spend the same time in both incentive zones. In the sexual satiety paradigm, sexually experienced males in the course of an ad libitum copulation session will ejaculate repeatedly (7 in ≈4 h) until reaching sexual satiety. Twenty-four hours after copulation to satiety, most males (≈70%) will not respond with sexual behavior when a receptive female is accessible. Pharmacological treatments can modify the features of the sexual satiety paradigm, the most important being the increase in the percentage of sexually satiated male rats exhibiting sexual behavior expression with a receptive female. If a drug treatment is administered prior to the copulation to satiety session, the result (percentage of animals exhibiting sexual behavior) will reflect the prevention of the establishment of sexual inhibition. If the treatment is administered 24 h after the sexual satiety session, the result will reflect a reversal. Figures modified from Canseco-Alba and Rodríguez-Manzo (2019).

The dopaminergic mesolimbic pathway is involved in the regulation of natural rewarding behavior (Kelley and Berridge, 2002), such as sexual behavior. An increase in DA concentration in the NAcc has been detected as soon as the male rat identifies the presence of a receptive female and during copulation (Pfaus et al., 1990; Robinson et al., 2001). In the DA neurons of the VTA, sex- and mating-related signals elevate c-Fos expression, a protein that can be used as a marker for neuronal activity (Balfour et al., 2004). The VTA’s neuronal activity increases during sexual engagement (Hernandez-Gonzalez et al., 1997). Every time they get access to a sexually receptive female, the vast majority of young, healthy male rats will act sexually in her presence. Moreover, the rewarding and reinforcing character of male rat sexual behavior is confirmed by the following observations: (i) male rats develop a conditioned place preference (CPP) for copulation (Tenk et al., 2009); (ii) males will cross an electric grid to have access to a receptive female (Bialy et al., 2019); and (iii) male rats will spend more time in the receptive female incentive area in the sexual incentive motivation test (Figure 2A; Bialy et al., 2019; Canseco-Alba and Rodríguez-Manzo, 2019).

Interestingly, in the course of an *ad libitum* copulation session with a single female, a male rat will repeatedly copulate with her until reaching satiety (seven ejaculations on average). The absence of sexual activity, when exposed to a new sexually receptive female 24 h after copulation to satiety is an expression of sexual satiety, a long-lasting sexual inhibition that can persist up to 72 h (Rodríguez-Manzo and Fernández-Guasti, 1994). It has been shown that during sustained copulation, DA levels in the NAcc remain elevated. Once the sexual activity ceases, DA gradually returns to basal levels (Fiorino et al., 1997). Also, once satiated, the male rat will spend less time in the female-incentive area of the sexual incentive motivation test immediately after its establishment (Ågmo et al., 2004) and 24 h later (Canseco-Alba and Rodríguez-Manzo, 2019) reflecting a reduction in sexual motivation (Figure 2B).

### Interaction between the endocannabinoid and the dopaminergic systems

**Figure 3:**
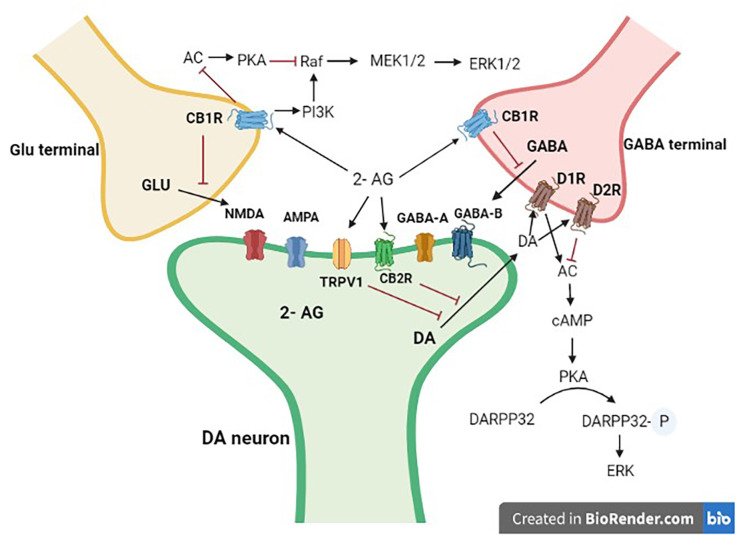
Interaction of mid-brain dopaminergic neurons with GABA and Glutamate terminals. Dopaminergic projections from the mid-brain VTA and the SN target postsynaptic glutamatergic and GABAergic axon terminals. 2-AG, 2 arachidonoyl glycerol; Glu, glutamate; DA, dopamine; GABA, γ-aminobutyric acid; CB1R, cannabinoid type 1 receptor; CB2R, cannabinoid type 2 receptor; D1R, dopamine type 1 like receptors; D2R, dopamine type 2 like receptors. Black arrows are stimulatory, whereas red ones are inhibitory.

Here, we provide an overview of eCB-DA interactions that are of increasing importance in motor control, reward processing, and psychosis. The ECS’s potential to interact with particular neurotransmitters like DA in various brain regions is probably what led to its involvement in brain functions (Figure 3), and other systems beyond DA and the classical neurotransmitters. Dopaminergic neurons that project to various forebrain regions, including the caudate-putamen and the NAcc/PFC complex, and whose cell bodies are found in the reticular formation of the midbrain (e.g., SNc and VTA) interact with the ECS. In these structures, both neuronal systems would exert a regulatory influence on numerous effector neurons, influencing activities like movement control and various cognitive functions, which are among the most important pharmacological effects of cannabis (Fernández-Ruiz and González, 2005; Gerdeman and Fernández-Ruiz, 2008).

#### Modulation of motor behavior

The basal ganglia contain most of the classical neurotransmitters, and many additional neuropeptides, which may participate in the modulation of motor activity. Some of the more important systems are GABA, glutamate, dopamine, and acetylcholine (Brady et al., 2012). The ECS components are highly expressed in the basal ganglia. The basal ganglia contain high levels of anandamide or 2-arachidonoylglycerol, as well as CB1R, the CBR type predominantly involved in the control of movement in healthy circumstances. The considerable modulatory role that the cannabinoid signaling system plays in the control of movement is logically explained by the presence of certain components of this signaling system in significant clusters of neurons and synapses within the basal ganglia circuits (Fernández-Ruiz, 2009).

The ECS has powerful actions on motor activity in animals (Geresu et al., 2016, 2019) and this effect is dose-dependent (Onaivi et al., 1990). Low doses of cannabinoids stimulated motor activity as evidenced by hyperlocomotion in intact animals and ipsilateral rotation in rats with unilateral 6-hydroxydopamine (6-OHDA) lesion of the substantia nigra. Large doses of cannabinoids reduced motor activity in a variety of behavioral tests and even produced strong catalepsy (Chaperon and Thiébot, 1999; Fernández-Ruiz et al., 2002). Similarly, motor activity was increased by low dosages of anandamide while it was decreased by moderate or high levels in rodents. Studies also showed that the CB1R antagonist rimonabant blocked the motor effects of CB receptor agonists, adding further evidence for the role of the ECS in controlling motor activity (Sulcova et al., 1998; Chaperon and Thiébot, 1999).

The ability of the ECS to modify the activity of the neurotransmitters that play a role in the regulation of basal ganglia function, particularly glutamate, GABA, and DA, is related to the motor effects that result from the activation or inhibition of cannabinoid signaling. The capability of eCBs to directly regulate the activity of these three important neurotransmitters is made possible by the identification of CB1Rs in a number of GABAergic and glutamatergic synapses within the basal ganglia and the presence of TRPV1 receptors in nigrostriatal dopaminergic neurons (Fernández-Ruiz, 2009). The effects of cannabinoids on the transmission of DA are often indirect and mediated by either postsynaptic or presynaptic processes. Such an indirect effect is made possible by the high density of CB1Rs in GABAergic, glutamatergic, or opioidergic projections found in close proximity to dopaminergic neurons. Studies also demonstrated that midbrain dopaminergic neurons, which lack CB1Rs, may yet synthesize and release eCB ligands from their somas and dendrites, enhancing the retrograde signaling function of these molecules *via* CB1Rs receptors in excitatory and inhibitory synapses (Fernández-Ruiz et al., 2010).

Studies have shown additional mechanisms for those eicosanoid-derived cannabinoids like anandamide, AM404, or *N*-arachidonoyl-dopamine that have some affinity for the TRPV-1 receptor, despite the fact that a majority of the cannabinoid effects on dopamine transmission appear to be GABA- and/or glutamate-mediated. These receptors, which are commonly found on sensory neurons and operate as molecular integrators of nociceptive stimuli, have also been discovered in dopaminergic neurons in the basal ganglia, allowing specific cannabinoids to directly affect dopamine function (Starowicz et al., 2007). Data suggest that the endovanilloid and DA signaling systems work in concert to govern a range of neurobiological functions, including movement control. Specific cannabinoids can directly control dopamine transmission by activation of CB2Rs and TRPV-1 receptors found in nigrostriatal dopaminergic neurons. Additionally, CB1Rs and dopaminergic receptors can form heteromers, which offers an additional mechanism for direct interactions between the two systems, in this case at the postsynaptic level. Cannabinoids’ interactions with the dopaminergic transmission in the basal ganglia are anticipated to have a significant impact on dopamine-related functions in these brain regions (García et al., 2016).

#### Modulation of motivational behavior

Mesocorticolimbic dopaminergic neurons control cognitive functions, motivate behavior, the central stress response, and the pleasure produced by reinforcers. Most of habit-forming drugs including cannabis derivatives produce their effect by altering the mesocorticolimbic DA transmission (Gardner, 2005; Fattore et al., 2008). Cannabinoid agonists produce euphoria, stimulate brain reward, decrease anxiety, motivation, and arousal while increasing emotionality (Lupica et al., 2004). Researchers agreed that glutamatergic and/or GABAergic innervations to the nucleus accumbens/PFC and VTA might serve as potential primary targets for the cannabinoid action in these processes and the ensuing DA changes (Pistis et al., 2001; Cheer et al., 2003).

Many brain areas that make up the brain reward circuitry are rich in components of the cannabinoid signaling system, especially the CB1Rs (Herkenham et al., 1991; Tsou et al., 1998). Cannabinoid agonists have been reported to increase mesolimbic dopaminergic activity as evidenced by elevated DA receptor density, DA release, and DA metabolism in various limbic structures (Fernández-Ruiz et al., 2010). They also enhanced the firing rate of mesolimbic dopaminergic neurons in the A10 region (González et al., 2005; D’Souza, 2007). Δ^9^-THC has been associated with increases in dopamine level (Braida et al., 2001) and neurotransmission (Oleson and Cheer, 2012) in the nucleus accumbens mesolimbic dopamine system. Pre-clinical studies also showed that VTA dopamine firing is increased by CB1 agonists (Melis et al., 2000). The DAT knockout mouse model of schizophrenia, which is characterized by high dopamine level in the striatum and nucleus accumbens, has been reported to have lower levels of anandamide in the striatum (Tzavara et al., 2006).

#### Role in neurometabolism

Mitochondria are a key organelle driving bioenergetic cellular processes for neuronal function and neurotransmission. Mitochondrial activity is involved in central control of energy balance and functional CB1Rs are located in brain mitochondria (mtCBRS), forming a basis for ECS signaling in mitochondrial neural and glial bioenergetic function (Hebert-Chatelain et al., 2014). MtCBR processes in neuronal network circuits and neurotransmission involve parvalbumin interneurons (Hebert-Chatelain et al., 2014). While studies have shown that brain regions rich with CBRs and mtCB1Rs regulate excitatory/inhibitory activity and balance (Hebert-Chatelain et al., 2014), the role of mtCBRs and (endo)cannabinoids in neuronal and glia cells enriched with mitochondrial function requires further characterization. For example, the effects of anandamide on reducing calcium sensitivity and perturbing membrane properties in mitochondria are independent of its target receptors and dependent on the mitochondria (Catanzaro et al., 2009).

D2-receptor (D2R)-mediated control of fast-firing parvalbumin-containing interneurons exerts a major influence on the prelimbic PFC (Fitzgerald et al., 2012). In the adult mouse PFC, parvalbumin interneurons fire more frequently when D2R is activated (Tseng and O’Donnell, 2007). These parvalbumin-containing interneurons of the PFC, whose dysfunction is linked to neuropsychiatric disorders, specifically schizophrenia, have a high D2R expression (Fung et al., 2010). Numerous neuropsychiatric conditions, including depression and schizophrenia, have been linked to dopamine homeostasis disruption during brain development. Studies found that D2R knockout mice had an ongoing increase of parvalbumin-positive neurons in the anterior cingulate cortex (Graham et al., 2015).

Mitochondrial bioenergetic dysfunction in inhibitory/excitatory input to parvalbumin interneurons can alter the excitatory/inhibitory balance in cortical microcircuits, leading to behavioral impairment in neuropsychiatric and neurological disorders (Inan et al., 2016; Maya-López et al., 2021) associated with dopamine dysregulation. Likewise, the change in D2R dendritic distribution and the decrease in mitochondrial mass of parvalbumin-containing interneurons in the medial prefrontal cortex of CB1R knockout animals (Fitzgerald et al., 2012) reinforces this notion. Specifically, in the CB1R knockout mice, there was a selective loss of expression and/or trafficking of dendritic D2R in the parvalbumin interneurons of mouse prelimbic PFC. Therefore, a disruption of dopamine input along with the inhibitory/excitatory circuits resulting from the deletion of CB1R could produce a deficit in parvalbumin interneurons of the prelimbic PFC (Fitzgerald et al., 2012). Perhaps it can be speculated that brain mitochondria, parvalbumin interneurons, endocannabinoids, and dopamine may be used as biomarkers in psychiatric disorders associated with dopaminergic dysregulation (Khadimallah et al., 2022).

### Role of the ECS in dopamine dysregulation

**Figure 4:**
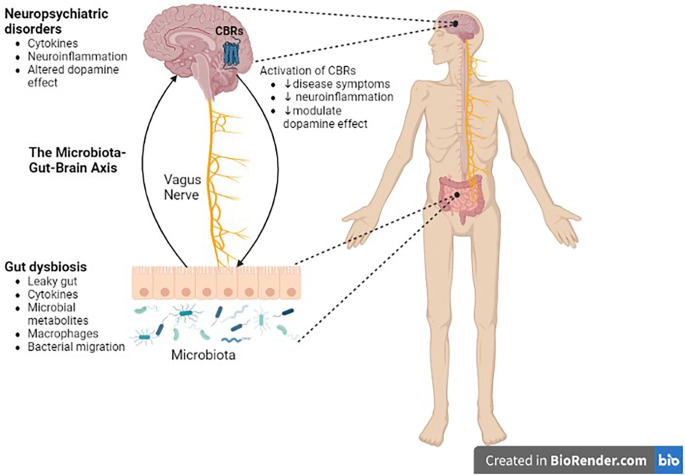
Role of the microbiota-gut-brain axis in CNS disorders. There is a bidirectional communication between the gut microbiota and the brain through the vagus nerve. Gut dysbiosis may play a crucial role in neural development, neurotransmission, neuroinflammation, and changes in neurotransmitter function, which in turn contribute to the pathogenesis of neuropsychiatric, and neurodegenerative diseases. Cannabinoid ligands targeting central cannabinoid receptors might be used in the treatment of Parkinson’s disease, drug addiction, or schizophrenia. CBRs, cannabinoid receptors.

The interaction of the ECS with dopaminergic system in the nigrostriatal or mesocorticolimbic structures of the brain has important implications in diseases associated with dopamine dysregulation in these brain structures. in situations when DA transmission is dysregulated, pharmacological modulation of these systems may help to normalize DA transmission and, as a result, relieve DA-related diseases (Figure 4). It is well understood that the ECS has an influence on classical neurotransmitters by retrograde signaling mechanisms, however, eCB-modulation of DA release and the impact on neural and behavioral processes require further studies.

### Role in parkinsonism

Dopamine receptors are abundant in the nigrostriatal dopaminergic pathway. There are direct and indirect pathways in the brain that start from the motor cortex and return to the motor cortex *via* the thalamus. PD occurs due to the disintegration of the nigrostriatal system. In PD neurons of the substantia nigra progressively degenerate. As a result, the amount of DA available for neurotransmission in the corpus striatum is lowered (Latif et al., 2021). Aberrant ECS signaling in the basal ganglia is suggested to be a culprit for PD and ligand-dependent modulation of the CBR could be one of the treatment approaches (Fernández-Ruiz, 2009; Wang et al., 2022). Indeed, the CB1R ligands have been proposed as a therapy for PD for symptomatic management (Geresu et al., 2016). Researchers also suggested the importance of CB1R agonists for the reduction of tremors caused by excessive stimulation of subthalamic neurons in PD patients (Sañudo-Peña et al., 1999) and to alleviate dyskinesia related to long-term levodopa treatment (Ferrer et al., 2003). In addition to the synthetic compounds, plant-derived cannabinoids (phytocannabinoids) having antioxidant properties showed promising effects to prevent dopaminergic neurons from degenerating in animal models of PD (García-Arencibia et al., 2007). CB2Rs also play a significant role in PD. Indeed, recent studies using mice with cell type specific deletion of CB2Rs on dopaminergic neurons revealed the role of CB2Rs in controlling motor effects (Liu et al., 2017; Geresu et al., 2019). To this effect, we and others reported promising effect of cannabinoid ligands against disease progression in wild type (Geresu et al., 2019) as well as MPTP-lesioned animals (Price et al., 2009; Geresu et al., 2019). These data collectively indicate the role of CBRs and their ligands in neuroprotection and preventing the progression of symptoms in patients with PD.

Results from clinical studies on the use of cannabinoids for the treatment of PD produced conflicting results. Cannabinoids may help with the motor symptoms of PD, according to some observational studies. In a study of 339 people with PD in the Czech Republic, 46% of participants said they had benefited from cannabis use; 31% said their rest tremor had improved, and 45% said their bradykinesia had improved (Venderová et al., 2004). Studies evaluating the effect of CBD in PD showed a reduction in total scores in the PD questionnaire and a significant lowering of non-motor symptoms (Zuardi et al., 2009). However, in another study, CBD administration produced no improvement in measures of motor and general symptoms in 21 PD patients (Chagas et al., 2014). An open-label study on 22 patients assessing motor exam 30 min after smoking cannabis also reported improvements in tremor, rigidity, bradykinesia, pain, and sleep (Lotan et al., 2014). In contrast, a few controlled clinical studies of cannabinoids reported no benefit for motor symptoms and mixed results regarding levodopa induced dyskinesia (Carroll et al., 2004; Mesnage et al., 2004). Despite contradicting results, the above data show that there is involvement of the ECS in PD. However, further research with randomized clinical trials should be done as evidence for targeting the ECS in the treatment of PD.

### Role in schizophrenia

Schizophrenia is a severe and persistent mental disorder characterized by cognitive, emotional, or behavioral symptoms. Despite the fact that the cause and pathophysiology of schizophrenia are still unknown, a substantial body of research suggests that changes in a number of neurotransmitter systems, including DA, are responsible for the pathophysiological processes that result in the manifestation of these symptoms. The DA system has drawn the greatest attention of these. The currently predominant view is that DA systems in schizophrenia might be characterized by an imbalance between subcortical and cortical DA systems (Guillin et al., 2007).

Investigating the interaction between the ECS and the dopaminergic system is also important in schizophrenia. According to studies, CB1Rs are found in substantial amounts in the dopaminergic terminals that are densely innervated in the brain areas involved in the pathophysiology of schizophrenia (Herkenham et al., 1991; Tsou et al., 1998). The stimulator effect of cannabinoids on DA transmission in the NAc (Müiller-Vahl and Emrich, 2008) and their inhibitory role on GABA and glutamate transmission may be responsible for causing or exacerbating psychoses (D’Souza, 2007). Genetic investigations have also suggested a connection between cannabis and the etiology of schizophrenia. These research linked a polymorphism in the CB1R gene with a higher risk of developing schizophrenia (Ujike et al., 2002; Müiller-Vahl and Emrich, 2008). In addition, according to the cannabinoid hypothesis of schizophrenia, higher CB1R density or endocannabinoid levels are observed in schizophrenic patients in cortical and subcortical (limbic) structures (Müiller-Vahl and Emrich, 2008).

Despite the evidence linking cannabinoids to schizophrenia, studies have also suggested that some cannabinoid-related substances may act as a cutting-edge treatment for schizophrenia (Voruganti et al., 2001; Bossong et al., 2009). There are several lines of evidence showing the antipsychotic effects of CBD and rimonabant. Animal studies demonstrated that the effect of the CB1R antagonist, rimonabant is related to alterations in DA (Tzavara et al., 2003) and glutamate (Tzavara et al., 2009) transmissions in cortical structures. Rimonabant did not vary from placebo in reducing both positive and negative symptoms in a placebo-controlled clinical trial conducted with patients suffering from schizophrenia and schizoaffective disorders, hence it did not appear to have any good benefits in clinical studies (Meltzer et al., 2004). With increasing interest in the use of cannabinoids for the treatment of psychosis, clinical studies revealed the potential of CBD in the treatment of patients with psychosis. The effects do not appear to be mediated by the activation of CB2Rs, as this phytocannabinoid has poor affinity for this receptor subtype. Thus, it is plausible to assume that it could be through modulation of the TRPV receptors or interference with endocannabinoid inactivation (Zuardi et al., 2006). In a double-blind RCT in 42 patients, oral CBD was found to be safe and led to a significant non-differential clinical improvement. Moreover, CBD also significantly increased anandamide levels, which was associated with clinical improvement (Leweke et al., 2012). Another double-blind parallel-group trial involving 88 patients with schizophrenia who were given oral CBD revealed lower levels of positive psychotic symptoms on the Positive and Negative Syndrome Scale (McGuire et al., 2018). In contrast, a recent double-blind randomized clinical trial (Boggs et al., 2018) found no benefit for 600 mg/day of CBD in comparison to a placebo. Although the involvement of ECS in schizophrenia has been described, results from preclinical studies using cannabinoid ligands are not entirely consistent. Only CBD and rimonabant were tested in clinical studies and hence additional controlled trials are required to confirm the therapeutic value of cannabinoids in schizophrenia.

### Role in reward and addiction

Preclinical researchers generally agree that dopamine has a significant role in the development of addiction. However, the specific role of dopamine in addictive behaviors is far from apparent, and only a small number of clinical research on the subject have been carried out in humans. There is a lot of convincing evidence that mesocorticolimbic dopamine has a role in reward and addiction in humans (Franken et al., 2005). The idea that pharmacological control of the ECS may be useful for treating addictive situations is supported by the ability of this system to affect DA transmission in corticolimbic regions. Previous studies demonstrated the potential of CB1Rs in this regard, the most-studied compound being rimonabant (Beardsley et al., 2009). Rimonabant impaired the perception of the reinforcing potential of different habit-forming drugs (Le Foll and Goldberg, 2005), indicating that motivational processes could be under a permissive control of CB1 receptor-related mechanisms. Addiction-inducing substances like opioids, cannabinoids, psychostimulants, alcohol, and nicotine boost DA release in several brain regions when taken acutely (Di Chiara et al., 2004). The ECS plays a crucial role in drug addiction. The mesocorticolimbic pathway, as well as areas of the brain related to decision-making, withdrawal symptoms, and relapse, express CB1Rs and CB2Rs (Maldonado et al., 2006; Liu et al., 2017; Silveira et al., 2017). Animal reward models highlight the role of CB1R in drug addiction, with CB1R agonists enhancing conditioned place preference (CPP). CB1R agonist-treated mice showed a greater preference for cues associated with ethanol and nicotine (Colombo et al., 2002; Valjent et al., 2002). Studies also showed that genetic and pharmacologic blockade of CB1R abolished drug-induced DA increases in rodents treated with Δ^9^-THC, nicotine, heroin, morphine, or ethanol (Cohen et al., 2002; Hungud et al., 2003; Soria et al., 2005).

Evidence also supports the involvement of CB2R in different aspects related to drug addiction. Psychostimulants are reported to produce differential effect in DAT-*Cnr2* conditional knockout (cKO) mice with selective deletion of CB2R in dopamine neurons and the effect is modulated by cannabinoid ligands (Canseco-Alba et al., 2019). Whilst cocaine, amphetamine, and methamphetamine produced robust CPP in both DAT-*Cnr2* cKO and WT mice; nicotine, at the dose used, induced CPP in WT but not in DAT-*Cn2* cKO mice. The enhanced hyperactivity induced by cocaine and nicotine was attenuated following pretreatment with the selective CB2R agonist JWH133 in the WT mice (Canseco-Alba et al., 2019). CB2Rs have also been shown to be involved in different aspects related to alcohol addiction. Mice that consume more alcohol had a reduced, whereas those with little preference showed no changes of *Cb2* gene expression in the ventral midbrain. Moreover, whilst the CB2 agonist JWH015 increased alcohol preference in mice subjected to chronic mild stress, the antagonist AM630 prevented the development of alcohol preference (Ishiguro et al., 2007).

Given how important CB1R is to the addictive qualities of the prototypical drugs of abuse, CB1R antagonists may be useful in the treatment of drug addiction. Rimonabant, a CB1R antagonist, has been shown to be successful in treating alcohol dependency in preliminary clinical studies and in clinical trials for the cessation of smoking as well (Manzanares et al., 2018). There are limited reports on the use of cannabinoid ligands for the treatment of drug addiction and dependency in humans. An experimental trial on a 19-year-old female with cannabis dependence showed that administration of CBD for 11 days (300 mg on day 1,600 mg on days 2–10, and 300 mg on day 11) rapidly decreased withdrawal symptoms (Crippa et al., 2013). In another study, Morgan et al. (2010) evaluated the impact of CBD on the reinforcing effects of Δ^9^-THC on addictive behavior and the findings suggested that CBD has a potential for the treatment of cannabis dependence. The acute modulation of the incentive salience of drug cues by CBD might possibly be generalized to the treatment of other addictive disorders. Currently, there is no effective treatment for drug abuse. Animal and a few clinical studies using CBD showed a promising effect of cannabinoids in the treatment of drug abuse, however, further research using different types of cannabinoids with new research strategies should be done to target the ECS for drug addiction.

The ECS has been implicated in the modulation of natural rewarding behaviors. For example, it has been demonstrated that eCBs have a role in the control of appetitive motivation and other aspects of food reward (Kirkham et al., 2002). Another highly rewarding behavior is sex (Bialy et al., 2019). The transition from sustained *ad libitum* copulation to sexual satiety, a behavioral inhibition, is linked to a fluctuation in extracellular DA level in the NAcc (Pfaus et al., 1990; Robinson et al., 2001; Canseco-Alba and Rodríguez-Manzo, 2019; Canseco-Alba et al., 2022). The ECS has been demonstrated to take part in the establishment of sexual satiety phenomenon, since a cannabinoid receptor antagonist is capable of preventing this inhibitory state when administered systemically (Canseco-Alba and Rodríguez-Manzo, 2014) or directly into the VTA (Rodríguez-Manzo and González-Morales, 2020) prior to the session of copulation until satiety. Thus, the specific blockade of CB1R in the VTA during copulation to satiety prevents the establishment of this sexual inhibition (Rodríguez-Manzo and González-Morales, 2020), suggesting that these eCB-mediated effects occur in this brain region by exerting a modulatory role on DA-induced natural rewarding behavior. In agreement with the well-documented evidence that eCBs are released “on demand” in response to repeated synaptic stimulation (de Fonseca et al., 2005), it is remarkable that a behavior taken to the extreme will set up mechanisms in order to keep homeostasis. Accordingly, the ECS components are expressed in brain regions involved in the regulation of copulation, such as the CB1R expressed in GABAergic and glutamatergic terminals within the VTA and NAcc (Lupica et al., 2004; Gardner, 2005). Supporting this notion, the administration of the eCB anandamide directly into the VTA reverses the sexual inhibition 24 h after copulation to satiety, an effect mediated by CB1 receptors (Canseco-Alba and Rodríguez-Manzo, 2016).

## Conclusion

ECS is a previously unknown system that has emerged as an indispensable component for regulating homeostasis and is involved in most cellular and biological processes. It is now known that the ECS is a vast signaling network that controls synaptic transmission throughout the brain and plays a crucial regulatory role in DA circuits (Fernández-Ruiz et al., 2010; Covey et al., 2017). Some DA-related neurobehavioral effects in the CNS are now understood to arise from interactions between the ECS and DA systems, including motor control or disorders (García et al., 2016; Geresu et al., 2016, 2019) and reward seeking or addiction (Gardner, 2005; Parsons and Hurd, 2015; Sagheddu et al., 2015; Zlebnik and Cheer, 2016). In the meso-cortico-striatal complex, DA-dependent and -independent mechanisms are associated with cannabinoid reinforcing effects, and drug-seeking responses associated with eCB/glutamate interaction, respectively (Spanagel, 2020). CBD is shown to operate through multiple receptor mechanisms to modulate brain DA, thereby reducing drug-intake and drug-seeking behavior (Galaj and Xi, 2021). Furthermore, in preclinical models, increasing eCB levels restores aberrant DA neuron activity in the ventral pallidum (Aguilar et al., 2015), and CB2Rs modulate midbrain DA neuronal activity and DA related behaviors (Zhang et al., 2014). Further support is provided by data implicating eCB modulation of DA release during reward seeking, interval timing, and active avoidance (Everett et al., 2021).

Drug addiction, PD, and other debilitating disorders caused by malfunctions of DA neurons still represent unmet clinical needs. The endogenous cannabinoid system may serve as a target for the development of new strategies for the treatment of CNS diseases associated with DA dysregulation. Cannabinoid agonist and antagonists might help in reducing the unwanted effects of conventional drugs used for the treatment of these disorders. However, care should be taken to minimize the psychoactive effects of cannabinoid agonists by designing selective drugs which may interfere with new molecular mechanisms involved in cannabinoid transmission. Currently, the evidence is nascent and too weak to recommend cannabinoid-based interventions for CNS disorders related to DA dysregulation. Research is only just beginning to determine the effect of cannabinoids for this application, and promising preclinical studies should be validated and complemented with clinical studies to claim the efficacy and safety of cannabinoids for the treatment of CNS diseases.

## Author contributions

BK reviewed the literature and provided a draft of the manuscript with AC-A providing the draft on the endocannabinoid and dopaminergic interaction in sexual motivation and satiety. EO and EE are involved in the design, conceptualization, and review of the article. All authors contributed to the article and approved the submitted version.
